# Cataract type and pupillary response to blue and white light stimuli

**DOI:** 10.1038/s41598-020-79751-8

**Published:** 2021-01-19

**Authors:** Manami Kuze, Kazuno Negishi, Toshiyuki Koyasu, Mineo Kondo, Kazuo Tsubota, Masahiko Ayaki

**Affiliations:** 1 Division of Ophthalmology, Matsusaka Central General Hospital, Matsusaka, Japan; 2 Department of Ophthalmology, Keio University School of Medicine, Tokyo, Japan; 3 Miwa Eye Clinic, Gifu, Japan; 4 Department of Ophthalmology, Mie University School of Medicine, Mie, Japan

**Keywords:** Medical research, Vision disorders

## Abstract

We evaluated the pupil reaction to blue and white light stimulation in 70 eyes with cataract and in 38 eyes with a selective blue-light filtering intra-ocular lens. The diameter of the pupil before stimulation was set as baseline (BPD) and, after a stimulus duration of 1 s, the post-illumination pupillary response (PIPR) was measured using an electronic pupillometer. The BPD showed no significant difference among three grades of nuclear sclerosis (NS). In contrast, the PIPRs differed significantly among the NS grades eyes including with and without subcapsular cataract (SC) and IOL eyes for white light (*p* < 0.05, Kruskal–Wallis test), but not for blue light. Subcapsular opacity did not affect the BPD or PIPR in all cataract grades for either light stimulus. The tendency of larger PIPR in the pseudophakic eyes than the cataract eyes for both lights, however significant difference was found only for white light (*p* < 0.05 for white light, *p* > 0.05 for blue light). Our study demonstrates retention of the PIPR for blue light, but not for white light in cataract eyes. We also confirmed that the pupillary response in pseudohakic eyes with a selective blue light-filtering intra ocular lens was greater than that in cataractous eyes for white light.

## Introduction

Yellowing increases in the aging lens, reducing transmission of light, especially short-wavelength light^1–3^. This arises from age-dependent accumulation of pigments in the crystalline lens that preferentially absorb blue light^4^. Progressive accumulation of lens pigments and insoluble crystalline aggregates leads to additional discoloration of the lens, inducing light scattering and loss of translucence^5^. Consequently, a cataract develops, and patients complain of blurred vision, glare and decreased visual acuity due to optical insufficiency. Attenuation of short-wavelength light with the aging lens may also lead to diminished non-visual light responses (e.g., pupillary light responses, melatonin suppression, and circadian entrainment) at this stage^6^. The most clinically significant cataract types are nuclear sclerosis (NS) and subcapsular cataract (SC)^7^.

Cataracts not only disturb vision but are also closely associated with systemic health problems, in terms of sleep, depression, cognitive function, and motor function, since cataract surgery is proven to be effective in improving these disorders^8–10^. Visual recovery and restoration of the transmittance of blue light may contribute to systemic and mental health improvements. Blue light-filtering and ultraviolet-filtering intra-ocular lenses (IOLs) are used in modern cataract surgery and while they have a comparable positive effect on sleep, there is concern that the blue light-filtering IOL might interfere with the circadian rhythm by reducing exposure to blue light. We previously reported an improvement in gait speed and sleep quality after cataract surgery using both types of IOL^11^. Brøndsted et al.^12^ reported that both the aging process of the natural lens and cataract formation could influence the photoentrainment of circadian rhythms, whereas pseudophakic eyes are not detrimental to circadian rhythm.

The pupillary light response is a reflex constriction of the pupil in response to an increase in ocular illumination, and is a basic clinical examination method for visual, autonomic and neurological function of the eye. Until recently, the pupillary light response has been thought to be driven by photoreception of rods and cones^13^. However, a novel photopigment, melanopsin, was discovered in the intrinsically photosensitive retinal ganglion cells (ipRGCs) of the inner retina in rodents^14^, and this category of RGC is recognized as playing a very important role in the pupillary light response^15–17^. The discovery of ipRGCs has added novel insights to the close relationship between circadian rhythm and general health, and their contribution to photoentrainment. Five different ipRGC subtypes in transgenic mice and two ipRGC subtypes in primates have been identified that differ in morphology and project to different brain areas^18^, and these inner retinal photoreceptors are thought to have the entire role of driving the post-illumination pupil response (PIPR)^19–21^. This sustained pupil constriction after a light stimulus matches the spectral sensitivity of the melanopsin pigment, thus making it useful as a direct biomarker of ipRGC function^20–22^.

However, the pupillary responses to different colored light stimulation have not been fully characterized in eyes with cataracts and IOLs. To the best of our knowledge, there has only been one study showing improvement of pupillary responses after cataract surgery in patients implanted with UV-filtering and blue light-filtering IOLs^23^. Moreover, how the type of cataract opacity, i.e., SC or graded NS, influences the pupillary response to blue light, i.e. photoreception of ipRGCs, has not yet been determined. The purpose of this study was to evaluate the pupillary light responses to blue and white light stimulation in eyes with various cataract types and in eyes implanted with a new selective blue light-filtering IOL.

## Results

A total of 115 patients were originally enrolled in this study. After the exclusion of seven patients due to incomplete examinations, the final analysis included 70 patients in the cataract group (mean [s.d.] age, 76.4 [6.4]; range, 65–88 years), and 38 patients in the IOL group (mean [s.d.] age, 76.2 [7.1]; range, 63–88 years). Patients in the cataract group were divided into three subgroups according to the grade of NS and each of these subgroups included patients with or without SC. Patient demographics and ocular parameters are summarized in Table 1.

**Table 1:** Patient demographics.

Diagnostic group	Cataract	IOL	*p-*value
No. of patients	70	38	
**Gender, n (%)**			
Female	39 (55.7)	16 (42.1)	0.211
Male	31 (44.3)	22 (57.9)	0.140
**Age, years (range, years)—all**	76.4 ± 6.4 (65–88)	76.2 ± 7.1 (63–88)	0.466
NS1	74.5 ± 7.2 (67–88)		
NS2	77.0 ± 5.8 (65–88)		
NS3	75.7 ± 5.2 (66–82)		
**NS grades, n (SC + / −)—all**	70 (35/35)		
NS1	16 (3/13)		
NS2	34 (21/13)		
NS3	20 (11/9)		
**BCDVA, logMAR**	0.33 ± 0.20	0.007 ± 0.14	< 0.01***

Data are mean ± s.d. unless otherwise indicated. ***Mann–Whitney U-test.

*BCDVA* best-corrected distance visual acuity, *IOL* intra-ocular lens, *logMAR* logarithm of the minimum angle of resolution, *NS* nuclear sclerosis (Emery–Little classification), *SC* anterior and/or posterior subcapsular cataract, *s.d.* standard deviation.

The results of pupillometry parameters for types of cataracts, with and without SC, are provided in Table 2. For each cataract NS grade, there was no difference in the BPDs (mm) between patients with and without SC (Mann–Whitney test). Likewise, the PIPR for white light and the PIPR for blue light did not differ between patients with and without SC in all three grades of NS. Overall, the presence of SC did not affect the BPD or PIPR for white or blue light.

**Table 2:** Pupillometry parameters in eyes with and without subcapsular cataract.

Parameters	NS grade	SC		*p*-value
		(–)	( +)	
BPD (mm)	NS1	2.99 ± 0.52	3.17 ± 0.12	0.388
	NS2	3.23 ± 1.02	3.22 ± 2.61	0.863
	NS3	3.49 ± 0.74	3.77 ± 0.69	0.448
PIPR white light (%)	NS1	3.91 ± 1.60	3.90 ± 3.01	0.779
	NS2	3.13 ± 1.66	3.54 ± 2.13	0.941
	NS3	2.85 ± 0.61	2.27 ± 1.56	0.062
PIPR blue light (%)	NS1	4.83 ± 1.82	5.57 ± 2.80	0.052
	NS2	5.64 ± 4.31	5.39 ± 2.22	0.519
	NS3	4.14 ± 1.87	4.51 ± 1.93	0.738

Data are mean ± s.d. *p*-values indicate the results of comparisons between SC− and SC + in each grade of NS (Mann–Whitney U-tests).

*BPD* baseline pupil diameter, expressed in mm, *NS* nuclear sclerosis (Emery–Little classification), *PIPR* post-illumination pupillary response, expressed as % constriction from BPD, *SC* anterior and/or posterior subcapsular cataract, *s.d.* standard deviation.

**Figure 1:**
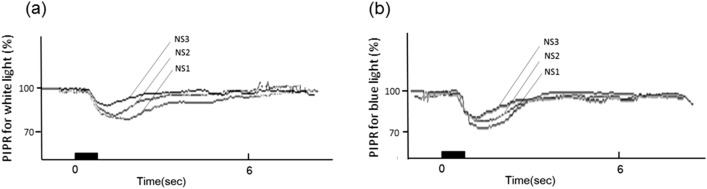
Representative pupillometry responses for the three grades of nuclear sclerosis (NS) after chromatic stimulus with white light (**a**), and blue light (**b**). Black bars represent stimulus duration. The vertical scale is expressed as % constriction from the baseline pupil diameter (BPD). (**a**) The responses obtained with white light show the initial transient constrictions followed by short-duration sustained constrictions. The more severe the grade of NS, the smaller the amplitude of pupil constriction observed. (**b**) For blue light, after the initial transient constrictions, the following sustained constrictions were stronger than those for white light. Smaller amplitudes of pupil constriction were observed in the more severe NS grades, as with white light.

Figure 1 shows the representative pupillometry responses for white and blue light in each cataract grade without SC. The responses obtained from white light show the initial transient constrictions followed by short-duration sustained constrictions, and the more severe the grade of NS, the smaller tendency of pupil constriction amplitudes observed (Fig. 1a). For blue light, after the initial transient constrictions, the following sustained constrictions were stronger than those obtained with white light. The tendency of smaller amplitudes of pupil constriction were also observed in the more severe grades of NS, as with white light (Fig. 1b).

**Figure 2:**
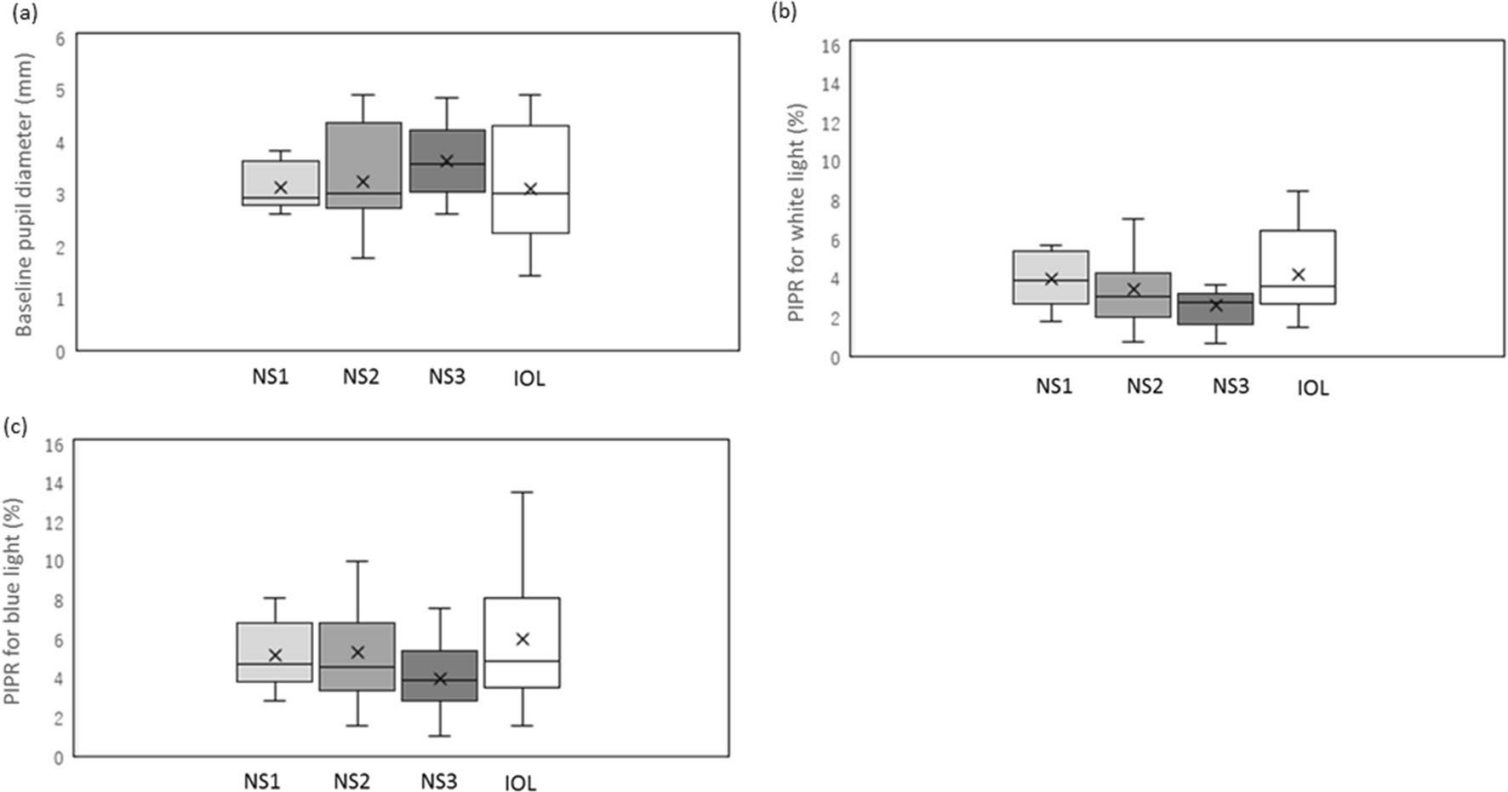
Results of baseline pupil diameter (BPD), the post-illumination pupillary response (PIPR), expressed as % constriction from BPD, obtained from eyes with various grades of nuclear sclerosis (NS) including with and without subcapsular sclerosis (SC) and IOL groups. (**a**) The BPR was not significantly different among the NS grades including with and without SC and IOL group (Kruskal–Wallis test, *p* > 0.05). (**b**) The PIPR for white light in the three grades of NS including with and without SC did not differ among the NS grades, but NS3 differed from IOL group (*p* = 0.037, Steel–Dwass test). (**c**) PIPR for blue light in the three grades of NS including with and without SC and IOL group. The PIPR for blue light was not significantly different among those groups (*p* > 0.05).

The results of BPD, PIPR among the grades of cataract including with and without SC and IOL are provided in Table 3 and Fig. 2, with each color light response assessed in relation to the NS grade of cataract including with and without SC (Table 3, Fig. 2).

**Table 3:** Comparison of pupillometry parameters between nuclear cataract and IOL .

Parameters	Lens status				*p*-value
	(95% CI)				
	NS1	NS2	NS3	IOL	
BPD	3.10 ± 0.64	3.22 ± 0.77	3.61 ± 0.71	3.08 ± 1.02	0.210
	(2.74–3.37)	(2.87–3.57)	(3.26–3.97)	(2.74–3.24)	
PIPR white light (%)	3.60 ± 2.0^a^	3.38 ± 1.94^b^	2.54 ± 1.13**	3.78 ± 2.12	0.038*
	(3.17–6.03)	(2.69–4.07)	(2.01–3.07)	(3.08–4.47)	
PIPR blue light (%)	5.09 ± 1.77	5.24 ± 2.85	3.92 ± 1.76	5.95 ± 3.26	0.091
	(3.82–6.36)	(4.21–6.27)	(3.02–4.83)	(4.88–7.02)	

Data are mean ± s.d. *p < 0.05, compared to IOL (Kruskal–Wallis test).

*CI* confidence interval, *BPD* baseline pupil diameter (mm), *IOL* intra-ocular lens, *NS* nuclear sclerosis (Emery–Little classification), *PIPR* post-illumination pupillary response, expressed as % constriction from BPD, *s.d.* standard deviation.

^a^*p* = 0.541.

^b^*p* = 0.878.

**p = 0.037 (vs IOL, Steel–Dwass test).

The BPD was not significantly different among NS grades and IOL, (*p* > 0.05, Kruskal–Wallis test; Table 3). Although the PIPRs for both white and blue light white showed the tendency of smaller amplitudes of pupil constriction in the more severe grades of NS (Fig. 1) and larger amplitudes in the IOL eyes (Fig. 2), only the PIPRs for white light between the NS3 grade (including with and without SC) and the IOL group were significantly different (*p* < 0.05, Kruskal–Wallis test, *p* = 0.037, Steel–Dwass test; Table 3, Fig. 2b,) while for blue light there was no such difference (*p* > 0.05, Kruskal–Wallis test; Table 3, Fig. 2c).

## Discussion

Light transmittance decreases with aging crystalline lenses and the magnitude of light-evoked pupillary responses are reduced^23^. In our study, the first to investigate the pupillary responses in eyes with various types of cataracts (NS and/or SC) and in eyes with selective blue-light filtering IOLs, we found that the PIPR to blue light in NS may maintain its amplitude despite the disturbance in transmission, as well as aging. In other words, the present results for patients with graded NS confirmed previous reports proposing an age-dependent compensatory mechanism^24^ with lens opacities, such as cataract, classified by indirect observations as a psychophysiological method. This result is consistent with the study by Rukmini et al.^25^ showing pupillary constriction responses to blue light are not selectively reduced in aging or in the presence of cataract, even in the yellowing lens of aged patients^1–3,26,27^. Daneault et al.^28^ also found the same phenomenon and reported that the magnitude of sustained pupillary constriction responses to blue light and green light stimuli was not reduced in aged subjects. A significantly greater reduction in PIPR for white light was recorded in eyes with severe nuclear cataract than in eyes with IOL, and this difference was not identified for blue light. Our results showed that the possibility of compensatory mechanism is more obvious with a blue light stimulus than other chromatic conditions, such as white light, because the spectral characteristics of opsin in ipRGCs has a peak sensitivity for short-wavelength light.

Light-induced melatonin suppression shows an age-related loss in sensitivity to short-wavelength light^29^. However, actual melatonin suppression does not proportionally decrease in aged subjects, contrary to experimental results tested with 460 nm blue light^30^. We speculate that a previously proposed hypothesis that a compensatory or adaptation mechanism against aging that causes the peak wavelength for melatonin suppression to shift to a longer wavelength^12,24^ might account for findings of the current study, although further investigations with non-cataractous controls would be necessary to confirm that.

Additionally, a rat model showed that density and dendritic arborization may not change with age^31^. Such adaptive changes could potentially occur in the retina itself, or in downstream targets of ipRGCs. Both visual photoreceptors and melanopsin contribute to sustained pupillary light responses^19,21^ but these photoreceptor types play different roles: rod-cone input is required for normal pupillary constriction at lower irradiances and in response to long-wavelength light, whereas melanopsin is required for normal pupillary responses to high-irradiance short-wavelength light^11,15,32–35^.

SC is an amorphous or fibrillary opacity on the lens capsule and induces light scattering^36^. On the basis of the Rayleigh phenomenon, the amount of scattering is inversely proportional to the fourth power of the wavelength^37^. The scattering of blue light increases with aging, which may help to preserve pupillary responses more than in younger individuals^36^. The present results indicate that SC might not reduce blue or white light sufficiently to affect pupillary responses in the same cataractous nuclear sclerosis, even though SC is a visually disabling severe pathology and often seen in older patients visiting ophthalmology clinics and patients with SC seriously suffer from photophobia and blurred vision.

Selective blue-light filtering IOLs may mimic rejuvenation of crystalline lenses with increased transmission of short-wavelength light and excitation of ipRGCs after surgery. Our present results confirmed that the pupil response obtained from selective-blue light filtering IOL was greater than that from cataractous eyes and are consistent with reports using full blue-light filtering IOLs^38–40^.

Pupillometry is a simple and easy clinical examination to evaluate the function of ipRGCs by using blue-light stimuli, since the human photoreception system is governed by ipRGCs, as historically shown in animals^14–17^. The pupillary responses for blue light may also be diminished in glaucoma^41,42^, Leber hereditary optic neuropathy^43^, age-related macular degeneration^22^ and retinitis pigmentosa^20^, as well as in cataract, as shown in the previous study^23,44,45^. In contrast, results in the P23H-1 rat, a model of inherited photoreceptor degeneration, showed decreased dendritic arborization but increased coexpression of Brn3a and melanopsin^31^, while the PIPR was restored in eyes with non-proliferative diabetic retinopathy indicating the resistant character of GCs and ipRGCs^46^. Another study revealed that both blue-blocking and neutral IOLs show a seasonal change, as well as having different characteristics in blue light transmittance affecting the sensitivity of ipRGCs, which is important for non-photosensitive visual function^44^. In our study, all of the pupil responses were recorded in the morning and during the fall to winter season. Thus, seasonal variations should not affect the present results.

The gradual increase in sensitivity of melanopsin-dependent responses with aging might follow a compensation for the gradual reduction in blue light to the retina, especially ipRGCs. These adaptive changes could occur locally in the retina or downstream from the ipRGCs^24,25^. Further studies are required to attest whether the adaptive conditions and pupil responses arise from ipRGCs.

Pupillary examination for cataract in the general population is clinically important, since cataracts and IOLs alter the transmittance of blue light which is directly linked to circadian control in the whole body. An electroretinogram is another examination to measure activity of human ipRGCs and we first described the clinical application of this technique in glaucoma patients^47^. Clinical examinations for ipRGCs are still being developed and further investigations are expected to achieve more information on human ipRGCs.

One of the limitations of the present study relates to the relatively small sample sizes of the groups and the different sizes of the cataract and IOL groups, however, the groups were statistically comparable because the enrollment of consecutive cataract or IOL cases was not biased and the appropriate statistical analyses were used. In this study, the cataract and IOL data were not obtained from the same patient, therefore we could not perform repeated testing from each patient. However it was suitable to use age-matched participants, since even the short period before and after surgery may affect pupillary responses in aged patients. Another limitation is that lens transmission was not measured in each case, as there is a significant reduction of light transmittance in aged yellowing lens^15,27,48^. However, there have been many studies exploring the spectral transmission in yellowing lens with aged subjects, for reference^1–3,26,27,49,50^. Further investigation with large case–control studies is necessary to confirm the influence of cataract type on pupillometry.

In this study, we did not observe a statistically significant difference between groups of PIPR for blue light. The large inter-subject variability could be the reason for this result. The PIPR for blue light is strongly influenced by ipRGC function, and we speculate that the following points could account for the inter-subject variability. First, there are genetic variations in the melanopsin gene expressed in ipRGC in humans. Second, there are large inter-individual phenotype variations in non-image forming effects of light^54–56^. Third, the stimulus condition of our study when using white light which may contain influence of cone photoreceptor^16,17,19,22,33^. Further study should be tested in future.

In conclusion, our study demonstrated retention of PIPR for blue light stimuli in eyes with all grades of nuclear cataracts, which supports the concept of a compensatory mechanism for photoreception in aged humans. We also confirmed that the pupillary response in pseudohakic eyes with a selective blue light-filtering IOL is greater than that in cataractous eyes for white light and equivalent for blue light.

## Methods

### Ethical approval and participants

This cross-sectional, case–control study adhered to the tenets of the Declaration of Helsinki and was approved by the Institutional Review Board of Mie University, Nabari Municipal Hospital and Matsusaka Central General Hospital. Written informed consent was obtained from all patients.

### Inclusion and exclusion criteria

Participants older than 55 years of age were recruited to the study at the Matsusaka Central General Hospital (Matsusaka, Japan) and Nabari Municipal Hospital (Nabari, Japan), from January to March 2016 and November to December 2017. Inclusion criteria were consecutive patients who underwent implantation of an IOL more than 2 months previously (IOL group) and age-matched outpatients with a clinically significant cataract, including NS (grade 1–3 on Emery-Little classification) and SC (cataract group). Patients were excluded if they were diagnosed with glaucoma, diabetic retinopathy, or an acute disease present for less than one month, identified by ophthalmic examination using slit-lamp, funduscopy, optical coherent tomography and fluorescein angiography. Pseudophakic patients were also excluded if they had major intra- or post-operative complications or a best corrected visual acuity poorer than 20/25.

### Pupillometry

The computerized chromatic pupillometry system consisted of two components: an infrared-sensitive charge-coupled device (CCD) array camera system with stimulus generator (FP-10000 II; TMI Inc, Tokorozawa, Japan) and a controller. We used two colored LED stimuli of white (463, 563 nm) and blue (470 nm). This system can generate a wide range of flash intensities, from 0.0001 to > 400 cd/m^2^ (− 4 to > 2.6 log) for white stimuli, and from 0.0001 to 400 cd/m^2^ (− 4 to 2.6 log) for blue stimuli. The camera with stimulus generator has an optical arrangement which can provide infrared illumination to highlight the pupil/iris border and also a controllable stimulation of the eyes. The CCD array camera system can record the changes in pupil diameter at variable stimulus duration, stimulation frequency and strength. The chromatic pupillometry setup was similar to previously reported^17,21,22^. The CCD system provides a continuous video signal output, which is sent to an external video frame capture device installed on a personal computer and analyzed using image capture software (View Shot TM; TMI Inc, Tokorozawa, Japan). With this technique, the video images are taken within a manually-set recording period. For evaluation, the pupillometer mathematically fits a circle on the border of the pupil. Then the average of five measurements is taken at a sampling rate of 20 Hz. In order to avoid the impact of circadian fluctuations in pupillary light responses on our findings^23,38^, all examinations were conducted during the day between 8 AM and 12 PM. In addition, to avoid the effect of seasonal variation, patients had their cataract surgeries from November to January, and their pupillometry were recorded from January to March^44^. Before the measurement, the participants stayed in a quiet room in mesopic conditions for 15 min^51^.

### Parameters from pupillometry

**Figure 3:**
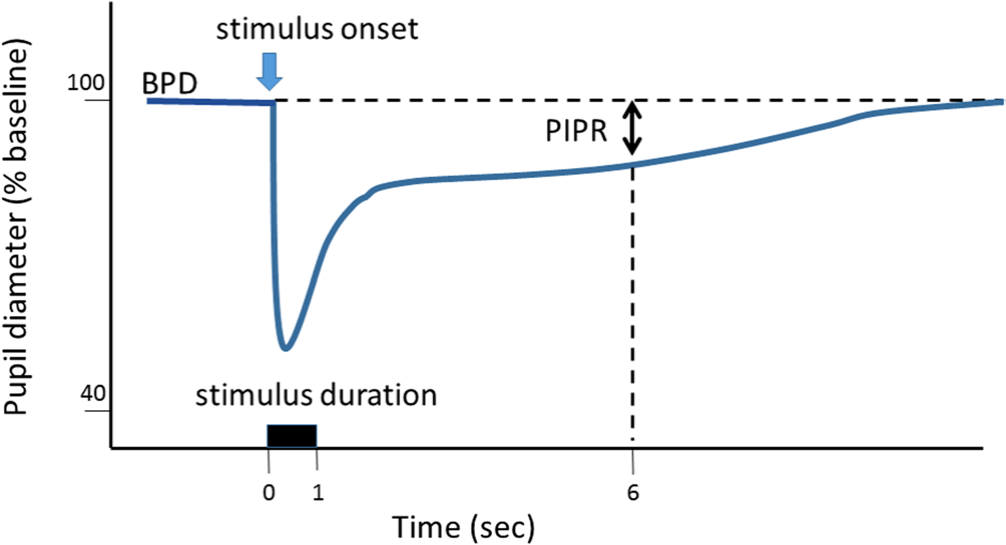
Parameters assessed for pupillary light response. The illustration shows the peak and sustained responses to light stimulation expected in healthy subjects. The vertical arrow indicates the stimulus onset. The duration is set as 1 s. The post-illumination pupillary response (PIPR) was determined as the value of constriction at 6 s after the light onset from baseline pupil diameter, and expressed as the % constriction from the baseline pupil diameter.

The measured values were analyzed according to protocols described elsewhere^21,22^. The examination consisted of two colors of light stimulations, white light (463 and 563 nm, both 400 cd/m^2^) and blue light (470 nm, 400 cd/m^2^). Briefly, the stimulus duration was 1 s and the recording period was 30 s per light stimulation. The interval for each stimulus was 60 s. An average of three recordings was set as one session for each different-colored light stimulus. An interval of at least 5 min was set between the two color stimuli to avoid the effect of the prior recording^21^. The baseline pupil diameter (BPD) was defined as the diameter over a 5-s period before light stimulation. The PIPR was recorded as the pupil diameter 6 s after the start of the light stimulus, following confirmation of a stable baseline diameter over a 5-s period. Since participants with a small BPD tend to display smaller PIPRs^45^, we used the PIPR as a percentage correction to remove this effect from the analyses. Thus, PIPR was calculated as the % constriction from the BPD; PIPR (%) = PIPR/BPD × 100. A schematic waveform of the pupillary light response and parameters is shown in Fig. 3.

### Ophthalmological examinations and surgical procedures

A cataract was diagnosed and classified as NS or SC under a fully dilated pupil with biomicroscopy to confirm a disturbed optical axis with significant opacity accompanied by the patient’s visual impairment. Eyes with cataract were divided into three groups according to the grade of NS—NS1, NS2 and NS3. Based on the presence of SC, eyes with anterior and/or posterior SC were categorised as with SC, and the rest as without SC. Routine ophthalmic examinations were performed by certified orthoptists and board-certified ophthalmologists.

**Figure 4:**
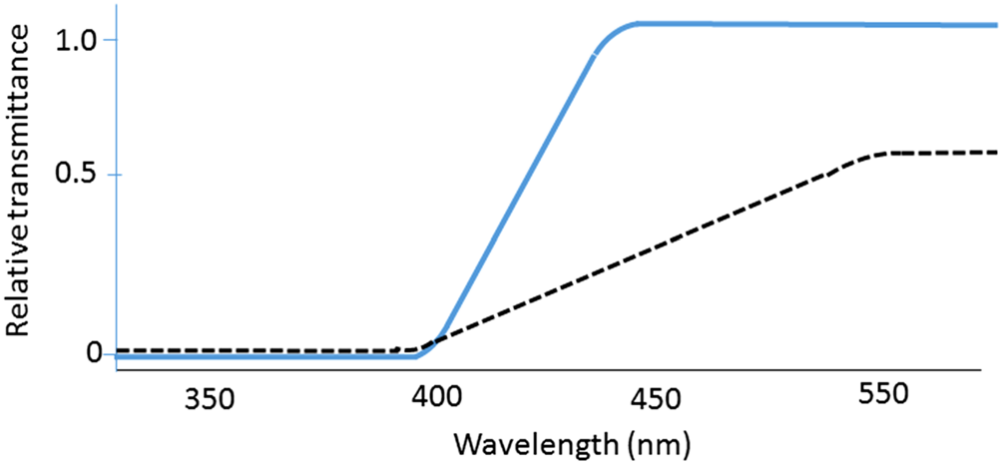
Spectral transmittance of the intra-ocular lens used in this study (solid line) and a 50-year-old human lens (dotted line), modified from the literature^52,53^. This intra-ocular lens (ZCB00V; Abbott Medical Optics Inc., Santa Ana, CA) was newly introduced to minimize retinal toxicity and adverse effects on circadian rhythms by filtering 100% of short-wavelength light under 420 nm and allowing longer-wavelength light, including 460 nm, to enter the implanted eye^57^.

Surgical procedures for cataract surgery and IOL insertion consisted of phacoemulsification and aspiration, followed by intra-capsular fixation of a posterior chamber IOL (ZCB00V; Abbott Medical Optics Inc., Santa Ana, CA) transmitting 95% at 480 nm. This IOL was newly introduced to minimize retinal toxicity and the adverse effects on circadian rhythms, by filtering 100% of short-wavelength light under 420 nm and allowing longer-wavelength light including 460 nm to enter the implanted eye (Fig. 4). All procedures were performed by experienced surgeons during the months of November to March. Anesthetics were topical and pre- and postoperative medication was identical between patients, including antibiotics and mydriatics. Anti-inflammatory ophthalmic solution (diclofenac) was used before, during, and for two months after surgery.

### Data analysis and statistical methods

Data from all tests were stored and the traces showing pupillary diameter were displayed; areas of data were selected for further analysis (Excel, Microsoft Corp. Redmond, WA). To avoid the influence of a variety of BPDs among individuals, ages and autonomic statuses, we calculated the PIPR as the % constriction from BPD, as described earlier^21,22^.

Data are presented as mean ± standard deviation and Mann–Whitney U tests were used to compare data between two groups. For comparisons involving three or more groups, the data were analyzed using Kruskal–Wallis tests. When Kruskal–Wallis test results were significant, Steel–Dwass testing for multiple pairwise comparisons was used to determine the individual differences among the NS and IOL groups. We calculated 95% confidence intervals (Cis). All analyses were performed with Excel Tokei for Windows^R^ Ver. 3 (SSRI Co. Ltd., Tokyo, Japan). All tests were two-sided, and *p*-values less than 0.05 were considered to be significant.
